# Fourteen years later: Reviewing the diagnostic criteria for behavioral‐variant frontotemporal dementia

**DOI:** 10.1002/alz.70604

**Published:** 2025-08-17

**Authors:** Olivier Piguet, Antonia J. Clarke, David Foxe, Nga Yan Tse, Her Teng, James R. Burrell, Sau Chi Cheung, James Carrick, Emma Devenney, Glenda M. Halliday, John B. Kwok, Carol Dobson‐Stone, Nicholas J. Cordato, Yun Tae Hwang, John R. Hodges, Rebekah M. Ahmed

**Affiliations:** ^1^ Brain and Mind Centre The University of Sydney Camperdown New South Wales Australia; ^2^ School of Psychology The University of Sydney Camperdown New South Wales Australia; ^3^ Faculty of Medicine and Health The University of Sydney Camperdown New South Wales Australia; ^4^ Neuropsychology Unit Royal Prince Alfred Hospital Camperdown New South Wales Australia; ^5^ Concord Clinical School Medical Education Centre Concord General Hospital The University of Sydney Concord New South Wales Australia; ^6^ St. George Clinical School University of New South Wales Sydney New South Wales Australia; ^7^ The Department of Aged Care St. George Hospital Sydney New South Wales Australia; ^8^ Calvary Community Health Calvary Health Care Kogarah Sydney New South Wales Australia; ^9^ Department of Neurology Gosford Hospital Gosford New South Wales Australia

**Keywords:** behavior, behavioral‐variant frontotemporal dementia, clinical presentation, cognition, survival

## Abstract

**INTRODUCTION:**

Diagnosis of behavioral‐variant frontotemporal dementia (bvFTD) requires the presence of at least three of six core features. The contribution of each criterion toward diagnosis, however, remains unclear. We aimed to determine the frequency and combinations of diagnostic criteria at presentation and their contribution to survival.

**METHODS:**

Consecutive patients with a stable diagnosis of bvFTD between 2010 and 2023 were included. Demographic and cognitive data were also collected.

**RESULTS:**

A total of 110 patients were identified. Frequency of diagnostic criteria ranged from 89% (apathy; empathy loss) to 14.5% (dysexecutive profile), and 78 patients met at least four criteria. Mean survival time from symptom onset was 10.4 years and was not affected by the number and combination of core diagnostic criteria (*P* values ≥ 0.274).

**DISCUSSION:**

Our findings show that the requirements for the cognitive criterion are disproportionately restrictive, which may delay diagnosis and access to relevant treatments and therapies.

**Highlights:**

- The comparative usefulness of core criteria for frontotemporal dementia diagnosis is unclear.
- We identified the most common profiles of core features at baseline.
- Most common feature profiles included apathy and/or empathy loss.
- Feature profiles were not predictive of survival.
- Cognitive criterion appears overly restrictive in its current form.

## 1 BACKGROUND

Behavioral‐variant frontotemporal dementia (bvFTD) is the most common syndrome associated with frontotemporal lobar degeneration (FTLD), accounting for between 50% and 70% of all cases of frontotemporal dementia (FTD).^1^ It is characterized by a progressive and pervasive decline in behavior and personality, and a variety of cognitive deficits, most commonly affecting executive function and social cognition.^2^, ^3^, ^4^, ^5^ A subset of patients may also present with, or develop, motor features associated with motor neuron disease or extrapyramidal motor disorders, such as corticobasal syndrome or progressive supranuclear palsy.^6^

Current classification and diagnosis of bvFTD is based on the 2011 consensus diagnostic criteria.^7^ These criteria focus on the presence of six core clinical features: five behavioral (disinhibition, apathy, loss of sympathy/empathy, compulsive behaviors, and hyperorality) and one cognitive (dysexecutive profile with preservation of episodic memory and visuospatial ability). Presence of these core features is generally gathered from various sources, including clinical investigations, cognitive evaluation, and caregiver‐based questionnaires.

The diagnosis can range in certainty between “possible,” “probable,” or “definite.” A “possible” diagnosis of bvFTD is indicated in the presence of progressive emergence and recurrence of at least three of the core clinical features, in any combination. A diagnosis of “probable” bvFTD is reached in the presence of documented decline in functional ability, as well as evidence of characteristic changes on structural or functional brain neuroimaging. Finally, a diagnosis of “definite” bvFTD is established in the presence of histopathological confirmation or identification of a causative gene mutation. Retrospective analyses of these diagnostic criteria have demonstrated good sensitivity and specificity for both young‐ and late‐onset bvFTD.^8^, ^9^

Fourteen years since their publication, it has become increasingly apparent that these diagnostic criteria are not without their limitations. For example, the operationalization of these criteria is not provided, nor is the timeframe for what constitutes an “early” change. The threshold for detection of behavioral change from a pre‐disease baseline requires more investigation, particularly across culturally and linguistically diverse groups. Similarly, the cognitive profile of bvFTD is far from uniform. Executive dysfunction is neither uniquely nor exclusively impaired in bvFTD.^10^, ^11^, ^12^ Additionally, a subset of bvFTD patients present with marked episodic memory deficits in excess of impaired attention or prefrontal retrieval processes.^11^, ^13^, ^14^, ^15^, ^16^, ^17^ Indeed, accumulating evidence has shown that the presence of marked episodic memory deficits does not exclude a diagnosis of bvFTD and that visuospatial deficits are more common than previously thought at various disease stages.^13^, ^14^, ^18^

Another challenge is that many of the core diagnostic features of bvFTD are also present in other neurodegenerative and non‐neurodegenerative conditions. Apathy, disinhibition, and reduced empathy are common features in primary psychiatric disorders (e.g., depression, bipolar disorder).^19^, ^20^, ^21^, ^22^ Similarly, language and episodic memory deficits, which are characteristic of Alzheimer's disease (AD), are present with variable severity in bvFTD.^14^, ^15^, ^23^ Further, given the progressive nature of dementia, clinical features tend to coalesce across disorders, presenting a challenge for clinicians when examining someone in the later stages of the disease.^24^

In light of this evidence, this study aimed to identify the most common clinical features and feature profiles among individuals diagnosed with bvFTD in the FRONTIER cohort in Sydney, Australia, since the publication of the diagnostic criteria, and determine their relations to clinical outcomes. Given the multiple permutations possible among this set of features, it is important to establish whether the presence or absence of certain features is indicative of a particular disease profile, including severity, progression, or survival.

We collated longitudinal data for 110 consecutive patients diagnosed with probable bvFTD at study entry using consistent operational thresholds for the behavioral and cognitive features. Using this approach, we aimed to determine the real‐world frequency of each core criterion and its combinations at presentation to evaluate the clinical applicability of the diagnostic criteria for bvFTD. In addition, based on these data, we suggest potential modifications to the current criteria to aid with early and accurate diagnosis, predict patient trajectories and aid eventual access to clinical trials.

## 2 METHODS

### 2.1 Participants

Participants were recruited from FRONTIER, the frontotemporal dementia research clinic based at the Brain and Mind Centre at the University of Sydney, Australia, between October 2010 and December 2023. Individuals were included in this study if: (1) they met the diagnosis for “probable” bvFTD diagnosis at the time of the first visit to the clinic (i.e., baseline) and (2) if the clinical diagnosis did not change over the course of the disease or until study dropout. In line with the criteria for a “probable” bvFTD diagnosis, all study participants exhibited atrophy in the prefrontal brain regions (orbitofrontal, dorsolateral, mediodorsal). Brain atrophy was established on visual inspection of the relevant magnetic resonance images in the coronal plane, using our previously validated rating scale.^25^ Individuals with a “possible” bvFTD diagnosis at presentation were, however, excluded given their variable underlying pathologies. Similarly, individuals with a mixed FTD‐amyotrophic lateral sclerosis phenotype, either at presentation or during the disease course, were also excluded from the study.

All participants underwent a neurological examination, a comprehensive cognitive assessment, structural brain magnetic resonance imaging (MRI), and genetic screening for the main FTD‐related genes. Collateral information was obtained from a carer (spouse, family members). Diagnosis was established by consensus among the examining neurologists, neuropsychologists, and occupational therapists. Exclusion criteria included a history of lifelong mental illness, alcohol or other substance abuse, significant head injury with loss of consciousness greater than 5 minutes, or limited English language proficiency to undergo a detailed cognitive assessment. Patients who scored < 50/100 on the Addenbrooke's Cognitive Examination‐III (ACE‐III) at baseline assessment, reflecting severe cognitive impairment, were also excluded.^26^

**RESEARCH IN CONTEXT**

1. **Systematic review**: The authors identified original and review articles using PubMed using keywords including “diagnosis,” “behavioral variant frontotemporal dementia,” “survival,” “behavior,” and “cognition.” These articles indicated that behavioral changes used as core criteria for the diagnosis of behavioral‐variant frontotemporal dementia are common. However, no study has established their prevalence, combination, and predictive value to disease survival.
2. **Interpretation**: Most patients endorsed four or five core criteria. Combinations of criteria were not predictive of disease severity or disease duration. Analyses further identified an overly restrictive cognitive criterion as currently defined, reducing its clinical applicability.
3. **Future directions**: We propose that the definition of the cognitive criterion be modified to be less narrow to increase sensitivity, which will improve access to treatment. Future research is also needed to determine whether a similar profiling exists in populations of different languages, different cultural backgrounds, and different socio‐economic backgrounds and education.

Ethics approval was obtained from the South‐Eastern Sydney Local Health District, the University of New South Wales, and the University of Sydney Human Ethics committees. In accordance with the Declaration of Helsinki, informed consent was obtained from all participants or their person responsible.

### 2.2 Clinical investigations

Changes in behavior (Criteria A–E) were assessed using one clinician‐based instrument: the Cambridge Behavioural Inventory‐Revised (CBI‐R; covering Criteria A, B, D, and E),^27^ and three informant‐based instruments: the Interpersonal Reactivity Index (IRI; covering Criterion C),^28^ the Frontal Systems Behavior Scale Family Rating (FrSBe; covering Criteria A, B, and C),^29^ and the Dimensional Apathy Scale (DApS; covering Criterion B).^30^ Potential discrepancies between the information arising from the clinician's investigations and the informant questionnaires were resolved during the visit to the clinic with the informant, with final decision (if necessary) made at the consensus meeting with the investigation team.

Changes in cognition (Criterion F) were measured with a range of instruments. The ACE, which evaluates the integrity of cognitive functions across five domains (attention and orientation, memory, verbal fluency, language, and visuospatial), was used as an overall cognitive screening measure. Patients completed the ACE‐III^26^ or ACE‐Revised.^31^ ACE‐Revised scores were converted to ACE‐III scores using validated algorithms.^32^

Additional tests were administered to measure the integrity of executive function. These included the Digit Span Backward (DSB) subtest of the Wechsler Memory Scale–Fourth edition,^33^ verbal letter fluency,^34^ Trail Making Test,^35^ and the Hayling Sentence Completion Test.^36^ Scores falling > 1.5 standard deviations below the mean of the control groups on these measures (or obtaining an age‐scaled score < 7 on DSB) were classified as “impaired.” Participants whose performance fell in the “impaired” range on two or more tests of executive function were classified as having a dysexecutive profile.

Disease duration was determined as the number of years elapsed from the reported onset of first symptoms to the date of assessment. Disease severity was calculated using the Frontotemporal Dementia Rating Scale (FRS),^37^ a 30‐item caregiver‐rated questionnaire which provides an index of functional impairment.

All participants underwent a whole‐brain, high‐resolution, T1‐weighted structural MRI brain scan on a 3T MRI scanner equipped with a standard quadrature 8‐channel head coil as previously described.^38^ Between 2010 and 2016, images were acquired on a Philips Achieva 3‐T scanner and from 2017, a GE Discovery MR750 scanner. To ensure the comparability of the T1‐weighted images, all scans were obtained using a standard 8‐channel head coil and harmonized protocols: 256 × 256 matrix, 200 slices, slice thickness 1 mm, 1 × 1 mm in‐plane resolution, echo time/repetition time = 2.6/5.8 ms, flip angle α = 8. Presence and location of brain atrophy were determined by visual inspection of images acquired in the coronal plane following our previously published protocol.^25^ In addition,^11^ C‐labeled Pittsburgh compound B positron emission tomography (PiB PET) imaging results were available for a small subset of patients (*n* = 9) who had participated in another study.

### 2.3 Statistical analyses

Data were analysed using IBM SPSS Statistics, version 29.0 (SPSS Inc.). Demographic and clinical variables (i.e., age at onset, years of education, disease duration, and ACE‐III scores) were compared across different diagnostic criteria using independent *t* tests. One‐way analysis of variance was used to compare these variables to the number and combinations of criteria met. Chi‐squared tests (*χ* ^2^) were used to analyze categorical measures (i.e., sex). Additional non‐parametric Mann–Whitney tests were carried out on the relevant demographic and clinical variables for each core diagnostic criterion to compare patients with and those without the criterion, and compare patients with familial or sporadic disease. Kaplan–Meier analyses were used to establish survival duration, and Cox regression analysis was applied to determine the contributions of diagnostic combinations, as well as other relevant demographic variables, as predictors of survival.

## 3 RESULTS

### 3.1 Demographic, clinical, and genetic variables

A total of 110 patients who met the inclusion criteria were identified. The demographic characteristics of the cohort are presented in Table 1. Genetic screening for the presence of a GGCCCC expansion in the *C9orf72* gene was performed for all samples where DNA was available (100/110 cases); additionally, next‐generation sequencing was performed on 64 samples. Collectively, these investigations for the presence of pathogenic gene mutations causative of FTLD in this cohort revealed positive findings in 24 patients. Twelve had a GGCCCC expansion in the *C9orf72* gene, six a *GRN* mutation, five a *MAPT* mutation, and one a *CHMP2B* mutation (Table 1). Another patient, who obtained a Goldman score of 1 (three first‐degree relatives affected across multiple generations),^39^ and thus a high suspicion for a genetic cause of disease, was also included in this group, although no confirmatory genetic testing was available. Among the remaining patients for whom DNA was unavailable, all had a Goldman score of ≥ 3 (i.e., no significant family history of younger‐onset dementia). These patients were therefore included in the sporadic bvFTD group for the analyses.

**TABLE 1:** Demographic characteristics of the bvFTD cohort.

	Total group bvFTD (*n* = 110)	Sporadic bvFTD (*n* = 85)	Genetic bvFTD (*n* = 25)
Sex (male:female)	72:38	57:28	15:10
Education (years)	12.3 ± 2.9 (8–25)	12.3 ± 3.1 (8–25)	12.1 ± 2.4 (9–18)
Age at disease onset (years)	57.9 ± 8.2 (32–74.8)	58.3 ± 8.2 (32–74.2)	56.4 ± 8.5 (37.5–74.8)
Age at assessment (years)	62.3 ± 8.5 (34.1–80.8)	62.6 ± 8.6 (34.1–77.7)	61.1 ± 8.1 (42.6–80.7)
Disease duration (years)	4.4 ± 2.6 (1–11.8)	4.3 ± 2.6 (1–11.8)	4.7 ± 2.6 (1.3–10.6)
ACE‐III total	76.8 ± 10.7 (51–94)	77.8 ± 10.7 (51–94)	73.2 ± 10.4 (52–94)
FRS	−0.45 ± 1.15 (2.49–3.09)	−0.48 ± 1.06 (1.92–3.09)	−0.32 ± 1.44 (2.49–2.58)
Genetic mutation	85:25	85	25
*C9orf72*	12	–	12
*GRN*	6	–	6
*MAPT*	5	–	5
*CHMP2B*	1	–	1
Other	1	–	1
*Post mortem* findings			
FTLD‐tau	8	6^a^	2^b^, ^c^
FTLD‐TDP	9	0	9
FTLD‐FUS	2	2	0
Alzheimer's disease pathology^d^	8	8	0
Other			0
Core criteria met (%)			
Three	32 (29.1)	22 (25.9)	10(40.0)
Four	51 (46.4)	38 (44.7)	13 (52.0)
Five	22 (20.0)	20 (23.5)	2 (8.0)
Six	5 (4.5)	5 (5.9)	0

*Note*. Values are means ± standard deviation (range). “Other” genetic reflects one individual with autosomal disease transmission across multiple generations but no genetic testing available. *Post mortem* findings.

^a^ Aging‐related tau astrogliopathy (ARTAG) with cerebrovascular disease and multiple infarcts.

^b^ FTLD‐tau GGT + FTLD Type A.

^c^ Mixed pathology of corticobasal degeneration with intermediate Alzheimer's neuropathologic change, limbic‐predominant age‐related TDP‐43 encephalopathy, and ARTAG.

^d^ With additional cerebrovascular disease (*n* = 2), cerebral amyloid angiopathy (*n* = 1), multiple infarcts (*n* = 1), and neocortical Lewy bodies (*n* = 1). FRS scores missing for 1 genetic and 4 sporadic cases.

Abbreviations: ACE‐III, Addenbrooke's Cognitive Examination III; bvFTD, behavioral‐variant frontotemporal dementia; FRS, Frontotemporal Dementia Rating Scale; FTLD, frontotemporal lobar degeneration; FUS, fused‐in‐sarcoma; GGT, globular glial tauopathy; TDP, TAR DNA‐binding protein.

In addition, *post mortem* pathological confirmation was available for 27 cases, including 11 of those with a gene mutation. Among the cases with *post mortem* results, 19 were found to have FTLD‐related pathologies: FTLD‐tau = 8 (including 2 with a genetic mutation), FTLD with TPD‐43‐immunoreactive pathology (FTLD‐TDP) = 9 (all 9 cases with a genetic mutation), and FTLD–fused‐in‐sarcoma = 2 (none with a genetic mutation). The 8 remaining cases all harbored AD pathology, some with additional vascular pathology (e.g., cerebrovascular disease, multiple infarcts) and one with additional neocortical Lewy body disease (Table 1).

Of the nine patients who also underwent PiB PET, scan results were negative for the presence of AD pathology in all cases. Within the PiB PET group, two cases had a *GRN* mutation and presence of FTLD‐TDP at *post mortem*, and the remaining seven were sporadic cases (i.e., no family history of younger‐onset dementia), including one who came to autopsy and showed evidence of FTLD‐tau at *post mortem*.

Altogether, neuropathological information was available for 46 participants (42%) in this cohort. Examination of the clinical presentations of the eight cases with non‐FTLD pathology did not show any significant differences in clinical features (age at presentation, disease, duration, cognition, disease severity at presentation). As such, these cases were retained in the subsequent analyses given the stability of their clinical diagnosis over the study period.

**FIGURE 1:**
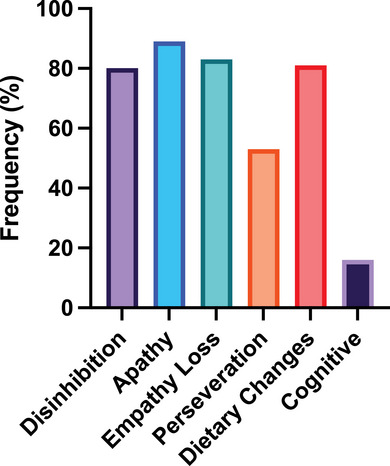
Frequencies of core diagnostic criteria for behavioral‐variant frontotemporal dementia. Each bar represents the frequency (%) of the core diagnostic criteria at presentation

Non‐parametric statistical analyses revealed no significant differences for any of the demographic and clinical variables (i.e., age at disease onset, years of education, disease duration, ACE‐III score, or disease severity) between patients with or without *post mortem* pathological confirmation. Similarly, no significant group differences were observed for these variables between patients with a sporadic or genetic form of the disease (all *P* values > 0.13), except for ACE‐III score, which was lower in the genetic group than in the sporadic group on average at presentation (73.2 vs. 77.8, *P *= 0.03; Figure 1).

### 3.2 Frequencies and profiles of core diagnostic criteria for bvFTD

The frequencies of the core diagnostic criteria for bvFTD ranged between 14.5% and 89.1% at presentation (Figure 1). The most common core criteria present were apathy or inertia (89.1%), followed by loss of sympathy or empathy (82.7%). Disinhibition, hyperorality, and dietary changes were also common and were observed in four out of five patients (80.9%). The least frequent diagnostic criteria was the neuropsychological profile (i.e., executive deficits with relative sparing of episodic memory and of visuospatial skills), which was found in only 16 patients (14.5%), followed by perseverative/stereotypical behavior (53%).

For each core criterion, we explored whether demographic characteristics differed between patients who met that criterion and those who did not (e.g., between those with and without apathy, etc.). Non‐parametric Mann–Whitney *U* tests showed that patients presenting with apathy or inertia had significantly lower ACE‐III scores (76.0 vs. 83.3; *U *= 359.5, *P* = 0.028) and lower FRS scores (–0.55 vs. 0.39; *U* = 306.5, *P *= 0.011) than those who did not. No other statistical differences were observed between patients exhibiting the target core criterion and those who did not (all *P* values > 0.05).

Almost half (51 patients, 46.4%) of the bvFTD patients met four of the core diagnostic criteria. Thirty‐two (29.1%) met three criteria, and 22 (20%) met five of the core criteria at presentation. The presence of all six criteria was rare and observed in only five patients (4.5%), consistent with the low proportion of patients meeting Criterion F (executive deficits with relative sparing of episodic memory and visuospatial skills).

**FIGURE 2:**
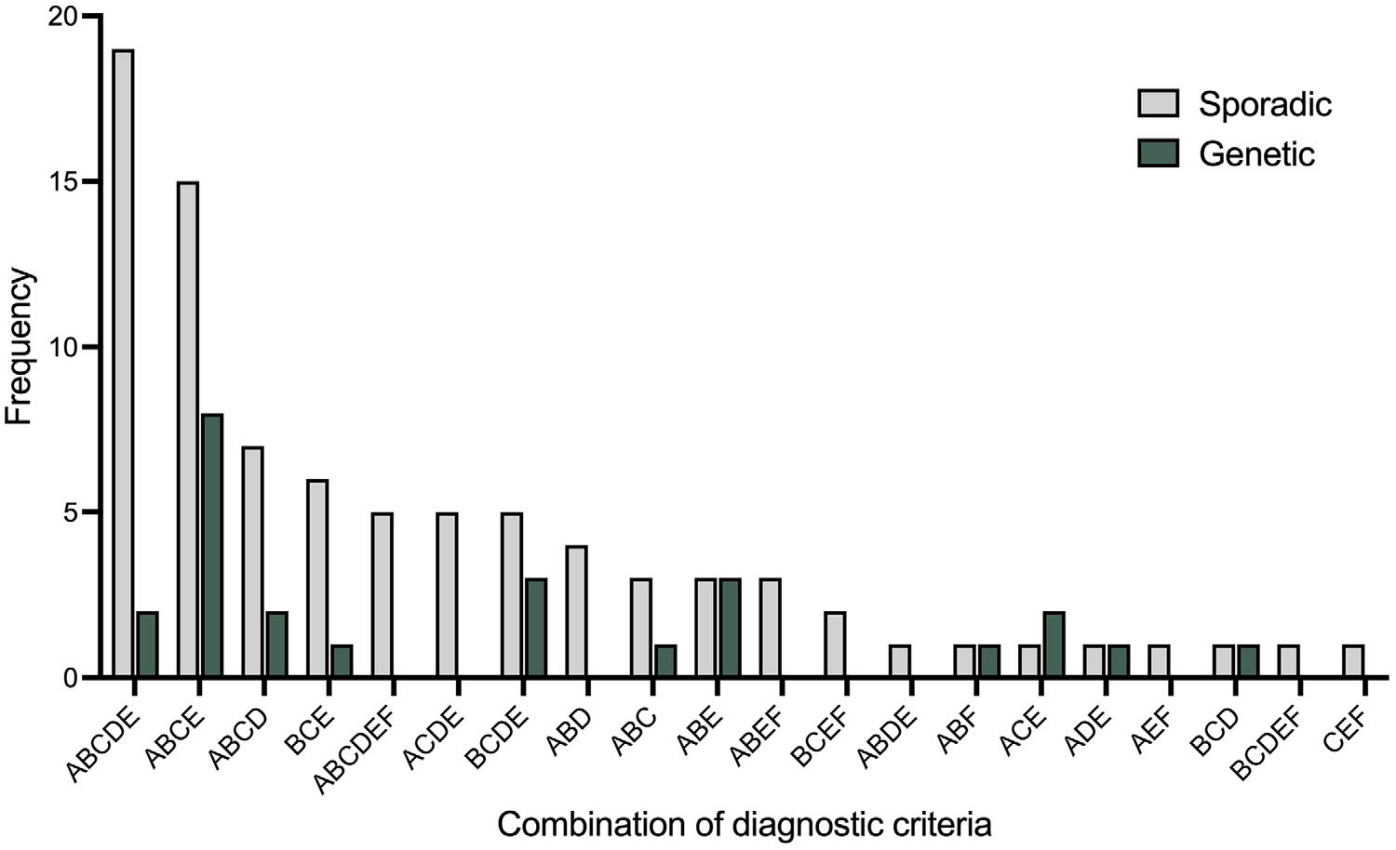
Frequencies of combinations of core diagnostic criteria for behavioral‐variant frontotemporal dementiain sporadic and genetic cases

Examination of the various possible combinations of core criteria revealed that the presence of Criterion B (apathy) was associated with Criterion A (disinhibition) and/or Criterion C (empathy loss) in 81% of cases (89 patients). In contrast, the combination of disinhibition and empathy loss without apathy was rare and found in only eight patients (7.3%). Also rare was the combination of eating disturbance and executive dysfunction, which was observed in only nine patients (8.2%), regardless of the presence of other core criteria (Figure 2; Table S1 and Figure S1 in supporting information). No specific pattern of associations was observed for Criterion D (perseverative/stereotypical behavior).

Additional examination of the presence/absence of the core diagnostic features in the subset of patients with a genetic presentation suggested similar profiles between the genetic and sporadic groups (Figure 2; Table S1, Figure S1). No formal statistical comparisons were performed on the frequencies of the core diagnostic criteria across the genetic groups, given the small number of cases. Similarly, these profiles did not change after excluding the cases with non‐FTLD pathology.

### 3.3 Cognitive criterion variability

**FIGURE 3:**
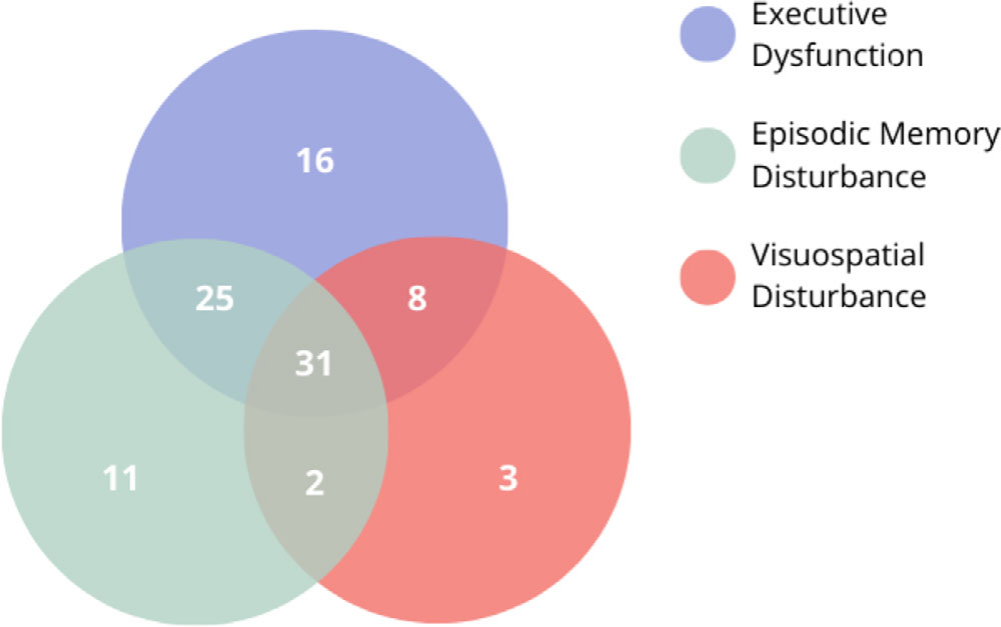
Core cognitive criterion (F) with and without concurrent exclusion features (episodic memory disturbance; visuospatial disturbance) in patients diagnosed with behavioral‐variant frontotemporal dementia

Although only 16 patients met the strict definition of Criterion F, almost 60% (64 patients) of the whole cohort experienced deficits in executive function in conjunction with one of the two exclusion criteria (episodic memory deficit [23%], visuospatial skill disturbance [7%]) and 31 (28%) exhibited all three cognitive features. Interestingly, 14 patients showed no cognitive features (i.e., a fully behavioral profile; Figure 3). Importantly, 69 patients (62.7%) experienced episodic memory disturbances, 11 (10%) of whom exhibited isolated episodic memory deficits only, that is, in the absence of executive and visuospatial deficits (Figure 2). In contrast, presence of visuospatial disturbances in isolation, or in combination with either memory or executive function disturbances (but not both) was rare (11.8%).

Relaxing the definition into a “broad” cognitive criterion (i.e., presence of dysexecutive profile with or without the additional cognitive requirements for spared episodic memory and visuospatial skills) impacted the frequency profile of core criteria. Applying this broad cognitive criterion definition, two thirds (68%) of patients exhibited four (*n* = 36) or five (*n* = 39) core diagnostic criteria, and 21 patients (19.1%) endorsed all six core criteria. Only 14 patients (12.6%) presented with three core criteria. No significant differences emerged between the number of criteria met at presentation using the strict or broad definition for the cognitive criterion and age at disease onset, education level, disease duration, ACE‐III score, or sex (all *P* values > 0.13). The various combinations of the core criteria using the “broad” cognitive criterion are shown in Table S2 and Figure S2 in supporting information.

### 3.4 Survival analysis

**FIGURE 4:**
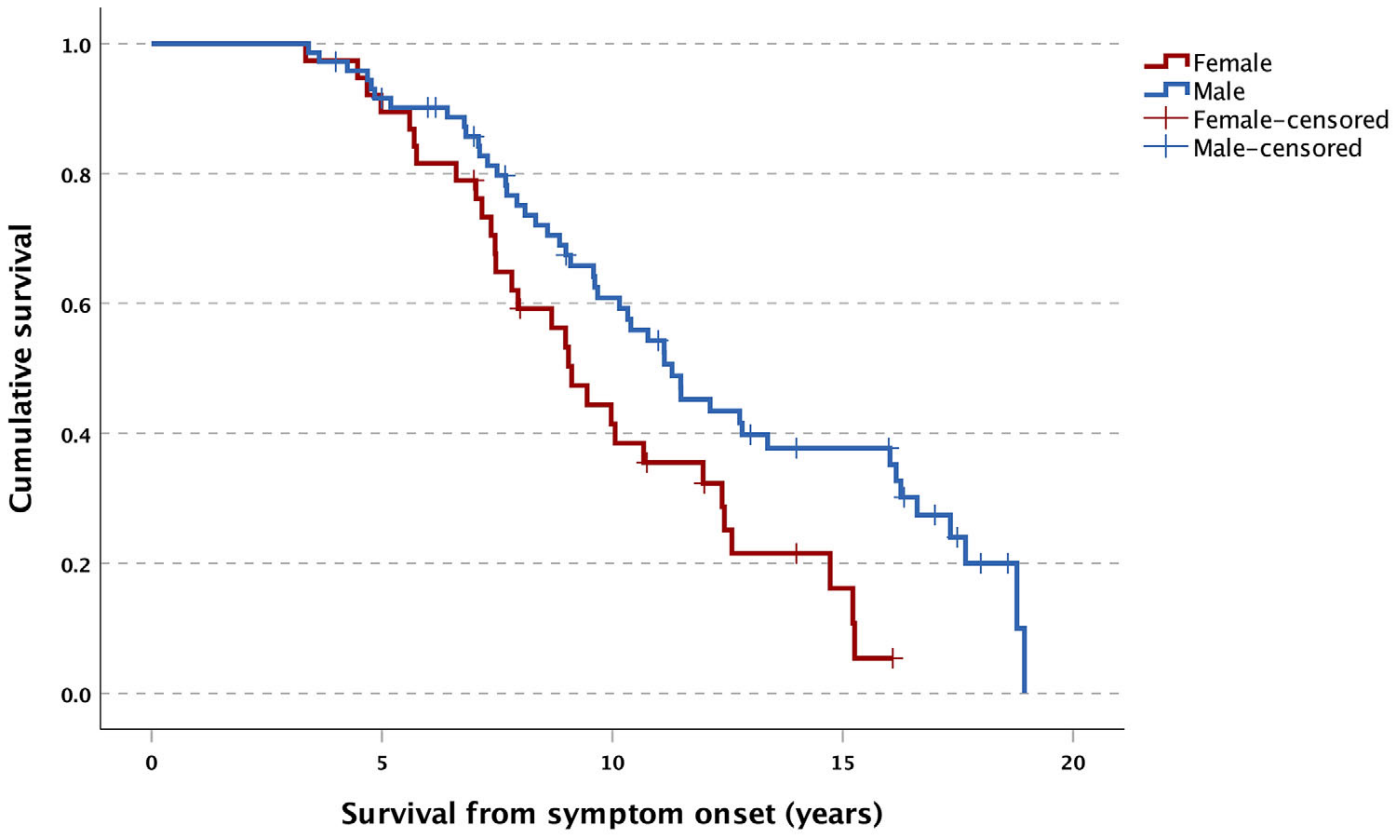
Mean disease survival from disease onset in male and female patients diagnosed with behavioral‐variant frontotemporal dementia

Kaplan–Meier analyses showed that the median survival time from symptom onset in this cohort was 10.39 years (95% confidence interval [CI]: 8.94–11.85 years), with females having a significantly shorter median survival compared to males (9.12 vs. 11.29 years; *X^2^* [1109] = 6.362; *P* = 0.012; Figure 4). Cox proportional hazard analyses indicated that the number of core diagnostic criteria met at entry into the study did not affect survival time (*X^2^* [3107] = 3.884; *P* = 0.274); nor did the five most common combinations of the core criteria (*X^2^* [5105] = 3.376; *P *= 0.642). Among other relevant clinical predictors, only longer disease duration (from symptom onset) was a significant predictor of shorter survival: hazard ratio = 0.786 (95% CI: 0.709–0.870*; P* < 0.001).

## 4 DISCUSSION

This study investigated the real‐world behavioral and cognitive profiles in a large cohort of well‐characterized patients clinically diagnosed with probable bvFTD, according to current criteria. Our investigations demonstrated the limitations of the cognitive criterion as presently outlined. Arguably, this criterion is at best unhelpful toward diagnosis, and at worst delays diagnosis and access to relevant treatment and therapy options. Our analyses identified the most common clusters of core criteria exhibited by patients at presentation. Importantly, none of these different presentations predicted disease trajectories or survival, or the genetic etiology.

Currently, a diagnosis of bvFTD is reached by meeting any three (or more) of the core diagnostic features. Importantly, only 16 patients (14%) met the cognitive criterion as currently defined.^7^ This criterion requires the presence of deficits in executive functions, in the context of relatively preserved episodic memory and visuospatial ability. Historically, executive dysfunction has been described as a hallmark of bvFTD, even early in the disease course.^40^ Although a majority of our cohort presented with executive dysfunction, this was commonly accompanied by other cognitive deficits—visuospatial, memory—which are currently exclusionary features. Indeed, when we applied a liberal approach to this criterion (i.e., executive dysfunction irrespective of memory and visuospatial performance), it was present in 80 patients (73%).

This finding has important clinical implications. The requirement for preserved episodic memory aimed to increase diagnostic specificity and the distinction from AD, in which episodic memory disturbance is considered a central and prototypical feature. This definition, however, has had unintended consequences with the belief that a “pure” or isolated, executive dysfunction is required for a diagnosis of bvFTD, discouraging clinicians from considering a differential diagnosis of bvFTD in the presence of executive *and* memory or visuospatial disturbances. Mounting evidence demonstrates that the requirement for a relative sparing of episodic memory in bvFTD is unduly restrictive. Patients with bvFTD may be impaired across various memory subdomains including working memory, episodic memory, learning, and recall and recognition memory.^13^, ^14^, ^38^, ^41^ Memory disturbance in bvFTD has been linked to pathological changes to extra‐hippocampal brain regions, including the prefrontal cortices.^42^, ^43^, ^44^ Importantly, however, disturbances of prefrontally mediated retrieval processes do not fully explain the memory deficits observed in this population.^42^, ^45^, ^46^ Indeed, 13 patients in our cohort demonstrated memory impairment in the absence of executive dysfunction. Similarly, disturbances in executive function is not exclusive to bvFTD and are seen in patients with underlying AD pathology, so‐called “frontal” AD.^12^, ^47^, ^48^

The requirement for a relative preservation of visuospatial ability poses a similar problem. This condition was designed to exclude dementias presenting with disturbances mediated by posterior brain networks, such as visuospatial and constructional skills.^17^, ^49^ Pathological changes in parietal regions tend to occur early in AD and remain comparatively preserved in early bvFTD.^17^, ^50^, ^51^ Nevertheless, these dementias may present with or without executive dysfunction;^52^, ^53^, ^54^ similarly, disturbances in visuospatial processing may also be present in bvFTD.^24^ In our cohort, almost half the patients had visuospatial deficits, in line with previous studies,^52^ including five without concomitant executive dysfunction. Importantly, none of these patients had AD pathology at *post mortem*. It is plausible that other cognitive disturbances, such as organizational approach, memory, apathy, and attention, may have influenced performance on visuospatial function assessment.^5^

Together, our results confirm that presence of memory and visuospatial deficits is common and does not preclude a diagnosis of bvFTD. We propose that Rascovsky Criteria F2 (relative sparing of episodic memory) and F3 (relative sparing of visuospatial skills)^7^—and similarly, the Diagnostic and Statistical Manual of Mental Disorders, Fifth Edition (DSM‐5) Criterion D (relative sparing of learning and memory and perceptual–motor function)^55^—be removed as core diagnostic features. Given the evidence of the breakdown in social functioning as an early and prominent feature of bvFTD, in contrast to other dementias (e.g., Shi et al.^56^), we further propose that disturbances in this domain should be added as another core criterion. Such an inclusion would increase the sensitivity and specificity of the diagnostic criteria as currently outlined, and would bring them in line with the DSM‐5, where decline in social cognition is considered separately from behavioral disinhibition and from loss of sympathy or empathy.^55^

In contrast to the cognitive criterion, behavioral core criteria were observed in ≈ 80% of patients, except for Criterion D (perseverative/stereotypical behavior), which was present in approximately half of the participants. These findings confirm the overwhelming behavioral nature of the syndrome. Interestingly, presence of apathy was generally associated with either disinhibition or empathy loss. indeed, the combination of disinhibition and empathy loss without apathy was rarely encountered, indicating the shared biological underpinning of these clinical features.

Importantly, presence or absence of a criterion was not modulated by clinical (disease duration) or demographic (age, sex, education) characteristics. Similarly, among the five most frequent combinations of core clinical features, neither the number nor the combination of criteria was accounted for by these variables or by ACE‐III score. Finally, the number, or combination, of the various core criteria present at initial assessment was not a significant predictor of disease survival. These findings support the broad applicability of these core diagnostic criteria across different contexts (e.g., variable education, age at onset, etc.).

Three aspects further demonstrate the value of the criteria for bvFTD. First, identification of abnormal behaviors is possible even in the absence of agreed or universal operationalization of these criteria. For example, no specified timeframe for “early” change exists, and the threshold for detection of behavioral change from baseline is likely to vary, particularly across culturally and linguistically diverse groups. Arguably, this issue requires more investigation, given the emerging notion of a prodromal period in bvFTD.^57^, ^58^ Second, identification of the behavioral disturbances can be reliably achieved using caregiver‐based questionnaires, especially when determining change from premorbid state. In a disease typified by reduced insight, caregiver reports are crucial to detect bvFTD early. Finally, establishing the presence of cognitive deficits is possible with common bedside neuropsychological tests reflecting “real‐life” clinical settings. Finally, median survival in our cohort was about 10 years from symptom onset, in line with existing literature.^59^, ^60^ Survival, however, was significantly shorter in women than in men, in keeping with a recent finding in sporadic bvFTD.^61^ This finding, however, contrasts with a recent report that found no sex difference in survival in a mixed sample of FTD patients presenting with behavioral or language changes.^62^ Notably, the authors did not report survival estimates for these syndromes separately.

### 4.1 Limitations

Arguably, this study is not without limitations. First, our data arose from a single specialized dementia research clinic. As such, our cohort may not necessarily be representative of the broad bvFTD population, which may influence the prevalence of some of the profiles reported here. Further, some patients may not be referred to specialty clinics because of variability in clinical features leading to misdiagnosis. It is also plausible, however, that it is complex patients that are referred to such specialist clinics because of their expertise in FTD and access to additional investigative resources that may not be available everywhere. In other words, unambiguous cases may have already been diagnosed upstream (e.g., by primary care physicians, or other specialists), without the need for more extensive investigations. Whether these cases exhibit different symptom profiles remains unknown.

Second, we used caregiver‐based instruments to determine the presence of behavioral features. Such instruments may be influenced by the caregiver's demographic characteristics and psychological state (e.g., stress),^63^ which may induce bias by either under‐ or over‐reporting behavioral features. Indeed, evidence indicates that male caregivers of people with dementia tend to minimize the severity of symptoms compared to female caregivers (e.g., Poisson et al.^64^). However, we have no reason to assume that if such a bias were present, it would not be randomly distributed across the cohort. In addition, as we only included patients with stable diagnoses, the bias risk may have been mitigated.

Finally, our study cohort was relatively homogeneous and comprised predominantly Caucasian/European participants. Certain cultural groups may have different thresholds for detection of behavioral changes, such as loss of empathy or disinhibition. This reinforces the need for comparative research evaluating behavioral changes across linguistically or culturally diverse patient groups in this population.

### 4.2 Conclusions and future directions

This study reports the real‐world frequency of the core diagnostic criteria for probable bvFTD in one of the largest cohorts of patients to date. Fourteen years after their publication, overall, these diagnostic criteria achieve their aim. Importantly, diagnostic profiles were not influenced by age, sex, education attainment, or disease duration, further supporting their broad applicability. An important limitation is, however, with the core Criterion F, which should be modified. We also argue that the addition of a criterion examining the integrity of social functioning is now warranted.

Despite this positive outcome, further investigations are needed to evaluate whether these findings are applicable to cohorts from culturally and linguistically diverse backgrounds. Similarly, research is also needed to determine the clinical profiles within pathologic and larger genetic subgroups of the disease, and their relations to neuroimaging findings. This in turn will help inform disease trajectory and ultimately aid in the development of pathology‐specific therapies.

## CONFLICT OF INTEREST STATEMENT

The authors declare no conflicts of interest. Author disclosures are available in the supporting information.

## CONSENT STATEMENT

All participants provided informed consent to take part in the study.

## Supporting information



Supporting Information

Supporting information
