# Supplementary material for: Fourteen years later: Reviewing the diagnostic criteria for behavioral‐variant frontotemporal dementia
Source: Alzheimers Dement. 2025 Aug 17;21(8):e70604. doi: 10.1002/alz.70604 (PMC12358242; doi:10.1002/alz.70604)
Supplement: Supplementary file 1 — Supporting Information [file ALZ-21-e70604-s002.docx]

**Supplementary Table 1:** Frequencies of combinations of core diagnostic criteria for bvFTD in sporadic and genetic patients applying the strict criterion F

|  | Sporadic (n = 85) | |  | Genetic (n = 25) | |
| --- | --- | --- | --- | --- | --- |
| Combination | Frequency | Percent |  | Frequency | Percent |
| ABCDE | 19 | 22.4 |  | 2 | 8 |
| ABCE | 15 | 17.6 |  | 8 | 32 |
| ABCD | 7 | 8.2 |  | 2 | 8 |
| BCE | 6 | 7.1 |  | 1 | 4 |
| ABCDEF | 5 | 5.9 |  |  |  |
| ACDE | 5 | 5.9 |  |  |  |
| BCDE | 5 | 5.9 |  | 3 | 12 |
| ABD | 4 | 4.7 |  |  |  |
| ABC | 3 | 3.5 |  | 1 | 4 |
| ABE | 3 | 3.5 |  | 3 | 12 |
| ABEF | 3 | 3.5 |  |  |  |
| BCEF | 2 | 2.4 |  |  |  |
| ABDE | 1 | 1.2 |  |  |  |
| ABF | 1 | 1.2 |  | 1 | 4 |
| ACE | 1 | 1.2 |  | 2 | 8 |
| ADE | 1 | 1.2 |  | 1 | 4 |
| AEF | 1 | 1.2 |  |  |  |
| BCD | 1 | 1.2 |  | 1 | 4 |
| BCDEF | 1 | 1.2 |  |  |  |
| CEF | 1 | 1.2 |  |  |  |

*Note:* ‘Combination’ refers to the core diagnostic criteria for behavioural-variant frontotemporal dementia.

**
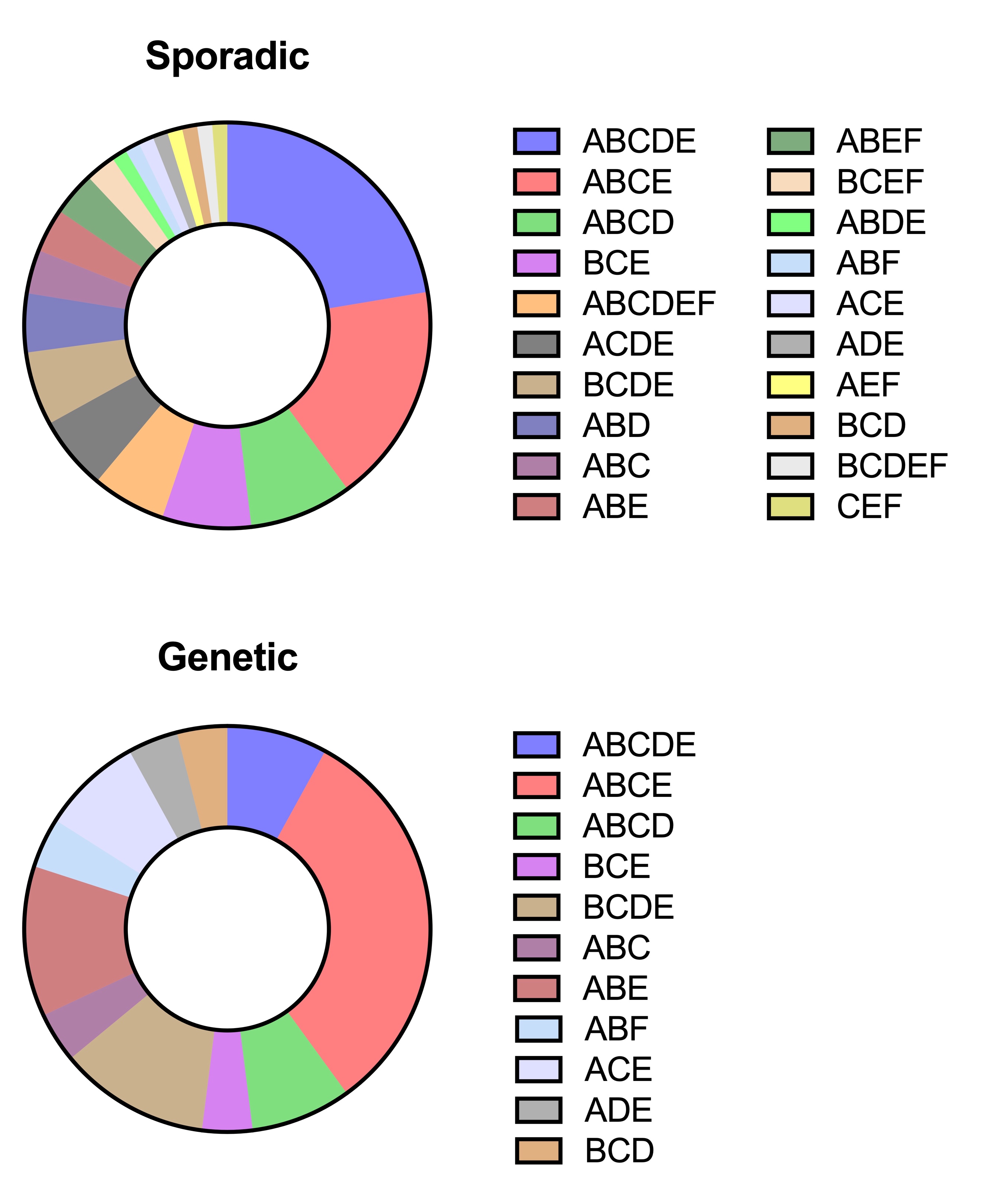
**

**Supplementary Figure 1:** Strict criterion F: Frequencies of combinations of core diagnostic criteria for bvFTD in sporadic and genetic patients.

**Supplementary Table 2:** Frequencies of combinations of core diagnostic criteria for bvFTD in sporadic and genetic patients applying a ‘broad’ criterion F.

|  | Sporadic (n = 85) | |  | Genetic (n = 25) | |
| --- | --- | --- | --- | --- | --- |
| Combination | Frequency | Percent |  | Frequency | Percent |
| ABCDEF | 19 | 22.4 |  | 2 | 8 |
| ABCEF | 10 | 11.8 |  | 7 | 28 |
| BCEF | 7 | 8.2 |  | 1 | 4 |
| ABCDE | 5 | 5.9 |  |  |  |
| ABCE | 5 | 5.9 |  | 1 | 4 |
| BCDEF | 5 | 5.9 |  | 3 | 12 |
| ABCDF | 4 | 4.7 |  | 2 | 8 |
| ABCD | 3 | 3.5 |  |  |  |
| ABCF | 3 | 3.5 |  | 1 | 4 |
| ABE | 3 | 3.5 |  |  |  |
| ABEF | 3 | 3.5 |  | 2 | 8 |
| ACDEF | 3 | 3.5 |  |  |  |
| ABD | 2 | 2.4 |  |  |  |
| ABDF | 2 | 2.4 |  |  |  |
| ACDE | 2 | 2.4 |  |  |  |
| ABDE | 1 | 1.2 |  |  |  |
| ABF | 1 | 1.2 |  | 1 | 4 |
| ACE | 0 | 0 |  | 1 | 4 |
| ACEF | 1 | 1.2 |  | 1 | 4 |
| ADE | 1 | 1.2 |  |  |  |
| ADEF | 0 | 0 |  | 1 | 4 |
| AEF | 1 | 1.2 |  | 1 | 4 |
| BCD | 0 | 0 |  | 1 | 4 |
| BCDE | 1 | 1.2 |  |  |  |
| BCDF | 1 | 1.2 |  |  |  |
| BCE | 1 | 1.2 |  |  |  |
| CEF | 1 | 1.2 |  |  |  |

*Note:* ‘Combination’ refers to the core diagnostic criteria for behavioural-variant frontotemporal dementia.


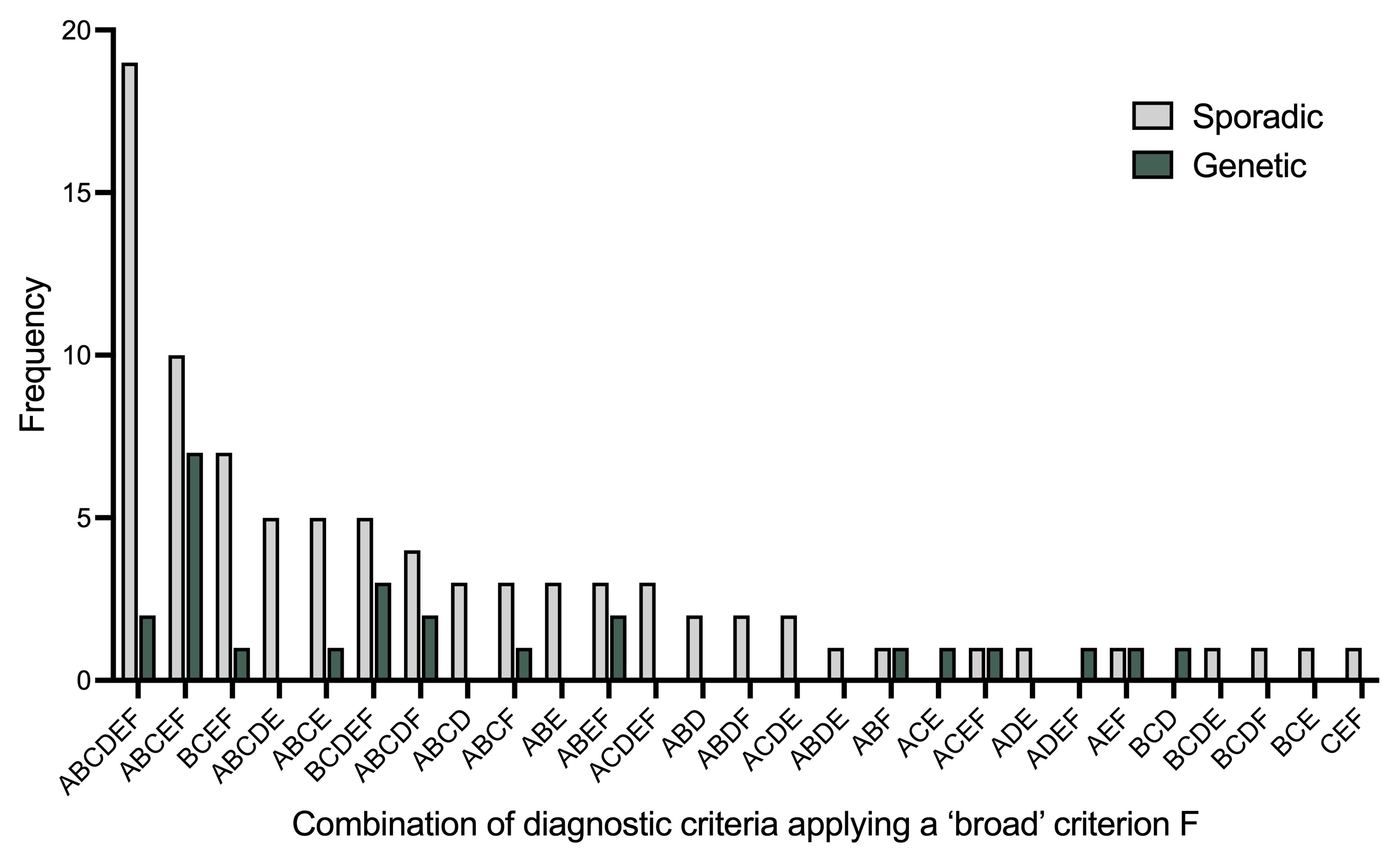


**Supplementary Figure 2:** ‘Broad’ criterion F: Frequencies of combinations of core diagnostic criteria for bvFTD in sporadic and genetic patients.
